# *SangsterLogP* - the largest publicly available dataset of *logP* values

**DOI:** 10.1038/s41597-026-07357-2

**Published:** 2026-05-07

**Authors:** Thalita Cirino, Giulia Caron, Giuseppe Ermondi, Larysa Charochkina, Igor V. Tetko

**Affiliations:** 1 Department of Molecular Biotechnology and Health Sciences, University of Turin, Turin, 10126 Italy; 2 V.P. Kukhar Institute of Bioorganic Chemistry and Petrochemistry, National Academy of Sciences of Ukraine, Kyiv, 02094 Ukraine; 3 Virtual Computational Chemistry Laboratory, Institute of Structural Biology, Molecular Targets and Therapeutics Center, Helmholtz Zentrum München, Neuherberg, 86764 Germany; 4 BIGCHEM GmbH, Unterschleißheim, 85716 Germany

## Abstract

We present *SangsterLogP*, the largest publicly available curated dataset of experimental *logP* values, comprising more than 23k unique molecules, with experimental *logP* values ranging from −3.8 to 11.7 (about 15.9 log units). The dataset originated from Dr. James Sangster’s comprehensive literature review of over 3k sources. We implemented a systematic curation workflow including a) *logD*-to-*logP* adjustment for ionised compounds and b) consensus-based residual analysis for outliers and duplicates removal. External validation using retrospective and prospective test sets demonstrated robust predictive performance (RMSE of 0.34 and 0.47 log units, respectively). *SangsterLogP* also substantially expands coverage of chemical space compared to the widely used legacy PHYSPROP database, including compounds in the beyond-Rule-of-5 domain. The fully annotated dataset, including experimental conditions and sources, is freely accessible via the Zenodo repository and on the Online Chemical database and Modelling Environment website

## Background & Summary

Lipophilicity, typically measured as the logarithm of the octanol–water partition coefficient (*log P*), is one of the most fundamental physicochemical descriptors in drug discovery and toxicology. It captures the balance between a molecule’s affinity for nonpolar versus aqueous environments, influencing *in vitro* Absorption, Distribution, Metabolism, and Excretion (ADME) properties such as solubility, permeability, protein binding, metabolic stability, and, ultimately, *in vivo* pharmacokinetics and toxicity^1^. Despite its long history and apparent simplicity, log P continues to play a central role in molecular design, guiding medicinal chemists through optimisation cycles and multi-parameter property trade-offs. *Log P* is, in fact, incorporated in most (if not all) rules of thumb, *e.g*. the Lipinski Rule of 5 (Ro5) and Veber rules, that accelerate compound prioritisation in very early and early drug discovery^2^.

Most drugs are partially or fully ionised at physiological pH. However, the interpretation of lipophilicity data becomes considerably more complex for ionisable compounds for at least two main reasons:

1. **Experimental ambiguity (*logP*** **vs** ***logD*)**. When a compound can ionise, its apparent distribution between octanol and water depends on the pH of the aqueous phase. Measurements made without strict pH control or without specifying conditions often yield *logD* values (the distribution coefficient)^3^, which corresponds to the combined partitioning of all species (ionised or not) at a given pH. Mixing *logP* and *logD* values within the same dataset can lead to large systematic errors, particularly for compounds ionised at experimental pH.
2. **Uncertain protonation state of the measured species**. Even when a nominal *logP* (neutral species) is reported, the actual measurement may involve partial ionisation if the experiment was performed at a pH near the compound’s pK_a_. Small deviations in pH or buffer capacity can thus shift the effective protonation state and bias the measured value. This issue is especially relevant for zwitterions, polyprotic molecules, and compounds with delocalized charge.

A critical review by Mannhold *et al*.^4^ noted that most of the *logP* predictive models have been trained and/or optimised almost exclusively on two legacy datasets (PHYSPROP^5^ collected by Syracuse Research Corporation mainly for environmental chemicals and the BioByte StarList^6^ collected by Leo and Hansch mainly for drug-like compounds), which strongly overlap, constraining their generalizability to the limited chemical space these collections represent. Although pharmaceutical companies continue to generate extensive proprietary *logP* datasets, such as Pfizer’s 96k-compound collection^4^ or Syngenta’s recent High-Performance Liquid Chromatography (HPLC) measurements of 27k molecules^7^, these remain inaccessible to the broader community. Several industrial datasets consist primarily of *logD* values measured at physiologically relevant pH^8^, which cannot be directly combined with *logP* data without systematic pH correction. Public repositories like ChEMBL provide substantial amounts of lipophilicity data, yet typically lack consistent curation and detailed reporting of experimental conditions, both of which are essential for robust model development. As a result, computational approaches continue to rely on a rather narrow pool of publicly available measurements, despite the existence of large but not publicly accessible experimental resources. The dataset described below, and named *SangsterLogP*, is designed to address this limitation by providing an openly accessible, well-curated, and machine-readable collection of *logP* values for roughly 24 000 unique molecules. It was achieved through systematic pH correction, outliers and structural redundancies (duplicates) removal *via* consensus-based residual analysis^9^.

Overall, *SangsterLogP* dataset spans a wide range of *logP* values (from −3.8 to 11.7 log units). Although the covered chemical space is predominantly (96.5%) Ro5-compliant, reflecting the historical bias of lipophilicity collections towards traditional small-molecule space, it also includes *logP* values for molecules belonging to the beyond-Ro5 (bRo5) chemical space. This latter encompasses macrocycles, peptides, protein degraders and other new modalities that are gaining prominence in drug discovery for their ability to address previously “undruggable” targets^10^ but whose physicochemical properties remain experimentally under-characterised, with large, validated lipophilicity datasets still lacking both in our work and in other benchmarking resources. Although the number of bRo5 compounds is smaller, their presence provides a valuable foundation for future expansion into this chemical space: an area of ongoing development in our group.

## Methods

### Dataset

The initial lipophilicity collection was compiled through Dr James Sangster’s extensive review of over 3,000 books and scientific articles, spanning his career up to his retirement in 2018. It comprised more than 30k unique molecules with at least one experimental lipophilicity (logP or logD) measurement, totalling more than 47k entries. It has undergone significant expansion since its initial publication^11^ and was continuously updated by Dr Sangster on a dedicated website until his retirement in 2018. The dataset was dynamically linked to the Virtual Computational Chemistry Laboratory (VCCLAB) *logP* predictor^12^, which redirected users to the Sangster website to display experimental values when available.

### Data preprocessing and ionisation-based classification

**Fig. 1:**
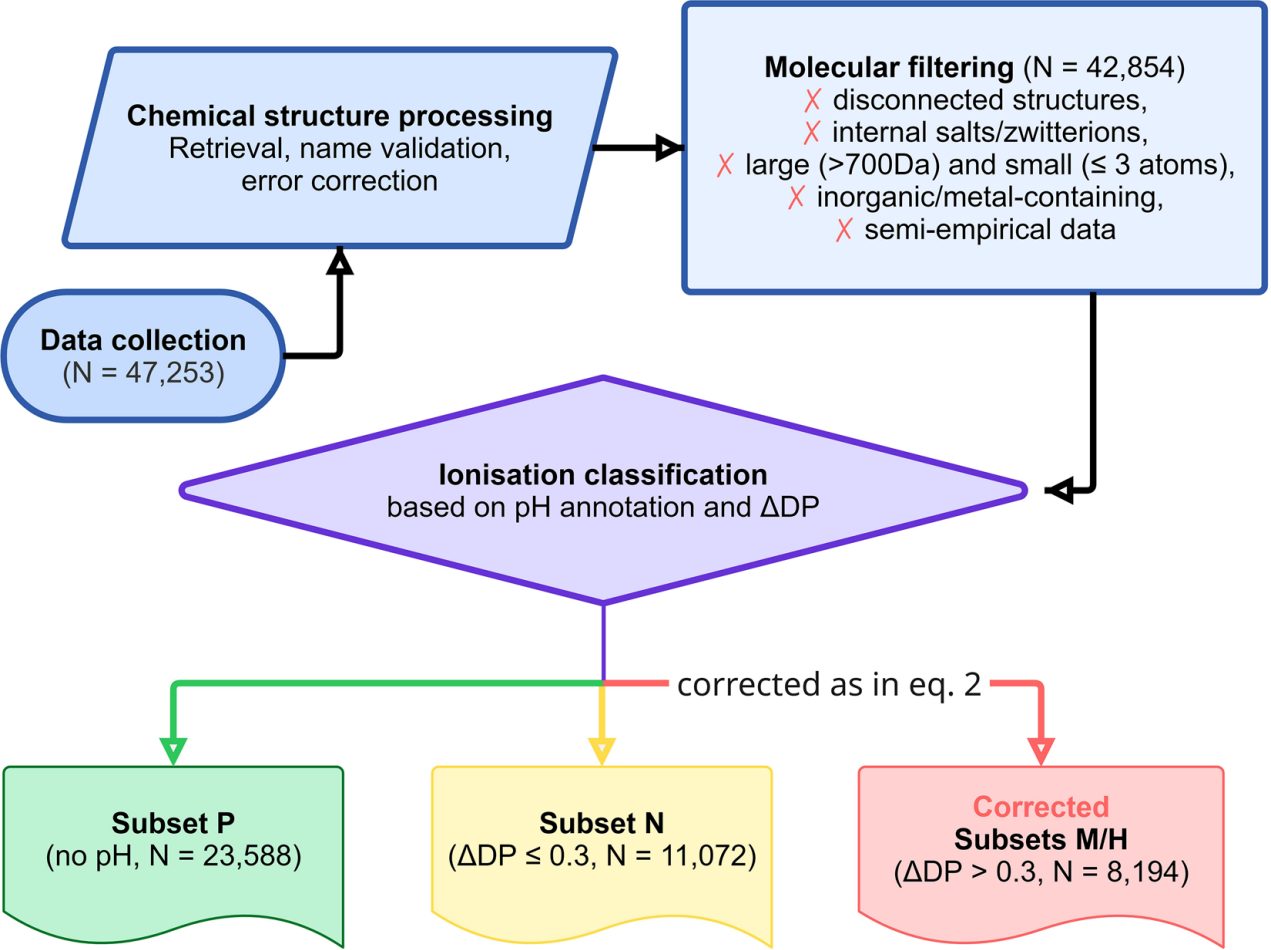
Preprocessing workflow from raw data collection through structure validation, molecular filtering, and ionisation -based classification into subsets: Subset P (no pH), Subset N (ΔDP ≤ 0.3), and corrected Subsets M/H (ΔDP > 0.3).

We developed a comprehensive curation workflow to transform the initial lipophilicity dataset into a high-quality curated repository suitable for computational *logP* modelling. All data processing, ionisation-based classification and curation steps were performed within the Online Chemical Modelling Environment (OCHEM, https://ochem.eu)^13^. Figure 1 illustrates our data preprocessing and ionisation-based classification workflow, which prepares the data for the next step (i.e., data curation).

First, we uploaded all data and searched various online databases for compounds without chemical structures, using the provided compound name or CAS Register Number (CAS RN). We corrected structural errors, including incorrect valence, non-typical valences, and aromaticity issues. Then, we systematically excluded several molecular categories to ensure compatibility with standard computational methods, including:

(i) disconnected structures (i.e., mixtures and salts);
(ii) internal salts and zwitterions;
(iii) large molecules (>700 Da, see below about this subclass) and very small molecules (≤3 atoms), retaining only drug-like compounds for analysis;
(iv) inorganic compounds;
(v) metal-containing compounds; and
(vi) records marked as obtained from semi-empirical calculations.

This filtering process reduced the dataset to 42,854 data points. We performed molecular identification and exclusion using SMARTS structural alerts^14^ implemented within OCHEM. These exclusions were applied for two main reasons: first, *logP* modelling of such compounds presents significant challenges using conventional computational methods; second, compounds in group (ii) remain ionised across the entire pH range, making *logP* determination impossible by its definition.

While compounds that were permanently ionised across the entire pH range were excluded as described above, 19,266 entries included measurements under conditions where the ionisation state depended on the experimental pH. To ensure that the dataset represents *logP*, we accounted for the influence of ionisation on the experimental records. Because *logD* is derived from *logP* and *pK*_*a*_, errors in either property propagate into the calculated *logD*. However, a previous study^15^ has shown that the largest contribution to the prediction error usually originates from inaccuracies in *logP* estimation itself, rather than from the ionisation-related shift (ΔDP). This is because this shift, calculated from the difference between *logD* and *logP*, tends to remain consistent across compounds that share similar ionisable groups. This explains why algorithms such as ALOGPS^16,17^, which were primarily developed for neutral compounds, could still provide reliable *logD* estimates in transfer-learning modes by identifying structurally similar compounds and adjusting for ionisation effects^15,18^. This reasoning provides the foundation for a systematic correction of reported *logD* measurements of major ionised species to *logP* done in this work. Therefore, we classified the pre-processed records into three subsets based on the confidence in *logP* values:

**Subset “original** ***logP*” (P, high confidence)** comprised either non-ionisable molecules or measurements under conditions where the neutral species were dominant. A high confidence was attributed to this subset since these measurements were provided by sources as *logP*.

**Subset “*logD*** **neutral” (N, medium confidence)** included experimental data with annotated pH at which the major microspecies were neutral or nearly neutral. The difference (ΔDP) between *logD* and *logP* was calculated according to Eq. 1:

1 $$\Delta {\rm{DP}}=\log {{\rm{P}}}_{{calc}}-\log {{\rm{D}}}_{{calc}}$$

where *logP*_*calc*_ was the predicted *logP* of the neutral species (equivalent to the maximum *logD* value found for the whole pH [0–14] range for non-zwitterionic molecules), and *logD*_*calc*_ was the predicted *logD* at the experimental pH. Both terms were obtained by ChemAxon software.

For subset **N**, ΔDP was ≤ 0.3, which represents approximately the experimental accuracy of *logP* measurements^3,19^. Therefore, these values were not corrected, as ΔDP was within the experimental error.

**Subsets “*logD*** **ionised” (M and H, low confidence)** comprised experimental data with annotated pH at which the major microspecies were (partially) ionised. For this subset, ΔDP > 0.3. These measurements were corrected to the corresponding neutral-species *logP*, according to Eq. 2:

2 $$\log {P}_{{adj}}=\log {{\rm{D}}}_{\exp }+\Delta {\rm{DP}}$$

where *logD*_*exp*_ was the reported experimental value. Of course, large ΔDP corrections could introduce higher errors. Therefore, this subset was further split into **M** (**moderately** ionised, 0.3 < ΔDP < 1) and **H** (**highly** ionised, ΔDP > 1).

This classification provides a practical balance between dataset size and reliability. Subset **P** contains the most reliable records, as these were explicitly reported as *logP* values, indicating that the original authors optimised or adjusted the measurements. Subset **N** has intermediate reliability; these values were initially reported as *logD* and reclassified as *logP* based on ChemAxon predictions verifying near-neutral measurement conditions. Subsets **M** and **H** carry the greatest uncertainty, as they require adjustment from *logD* to *logP* using ΔDP corrections, with uncertainty increasing proportionally with the magnitude of the correction.

The conversion of *logD* to *logP* values was done by predictions obtained with the ChemAxon software and a custom Python script that implements the procedures described above^20^. Considering that the prediction of *pK*_*a*_ is a difficult task and novel methods constantly appear, it is possible that newer versions of ChemAxon or other tools can provide better conversion and improve data and models developed using them. The provided annotation of the data file in which we report adjustments allows a straightforward use of such methods.

### Data curation

We performed data curation to remove duplicates and outliers (*i.e*., records whose predicted–experimental residuals were statistically improbable based on a consensus-model analysis). To identify such outliers, we used a consensus-based residual analysis previously shown to be effective for error detection in large experimental datasets^9^.

In this study, this analysis relies on a consensus model built from a diverse ensemble of machine-learning (ML) methods. We selected six of them available in OCHEM: two Natural Language Processing methods (Transformer CNN^21^ and Transformer CNF2^22^) and two methods from Keras Graph Convolution Neural Networks^23^ (Attentive Fingerprints^24^ and ChemProp^25^) in the pool of representation learning methods. Amid descriptor-based methods, we selected a combination of an extended set of Estate descriptors^26^ with DNN^27^ and OSMORDRED^28^ descriptors with CatBoost^29^. The rationale behind the selection was to combine different algorithms to maximise the consensus model diversity. These methods also contributed to the winning and runner-up models of Tox24 challenge^30^, thus showing their high prediction accuracy. For each data point, a consensus *logP* prediction was obtained by averaging the predictions from the six individual methods, and the standard deviation across predictions was used as a distance-to-model metric^31^, which provides a reliable estimation of prediction uncertainty. By fitting a mixture of Gaussian distributions to the residuals, we estimated the probability of each prediction being generated by the distribution. Records with large residuals but low uncertainty are unlikely to be generated by such distributions (*i.e*., *p* < 0.001), and so those outliers were flagged for removal since they likely represent experimental errors or correspond to experiments with inadequate pH for measuring *logP*^9^.

Duplicates handling followed a two-tiered procedure: (i) when multiple entries reported identical lipophilicity values for the same molecule, we retained the earliest record; and (ii) for remaining duplicates with differing values, we kept the record with the smallest residual relative to the consensus prediction. This procedure was applied twice for each subset.

**Fig. 2:**
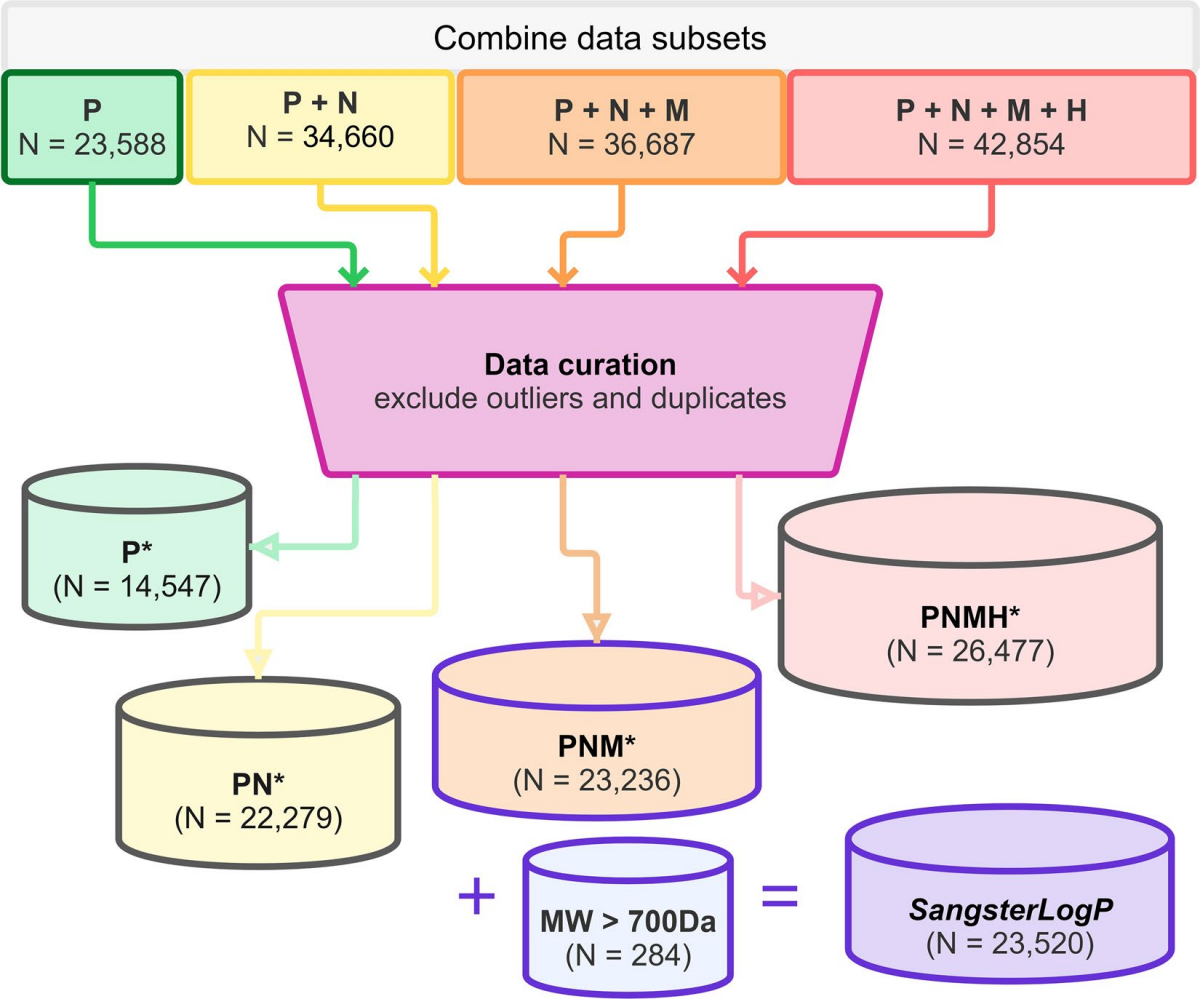
Four parallel curation pipelines process progressively inclusive datasets: P alone; P + N; P + N + M; and P + N + M + H. Each pipeline applies consensus-based outlier detection and duplicate removal, yielding curated datasets denoted with asterisks (P*, PN*, PNM*, and PNMH*). Colours indicate confidence levels: green (high), yellow (medium-high), orange (medium-low), and red (low). An additional set of large compounds (MW > 700 Da) was incorporated into the PNM* dataset to generate the *SangsterLogP* dataset.

We applied this data curation, combining outliers and duplicates retrieval in parallel to four subsets: (1) P alone, (2) combined P + N, (3) P + N + M, and (4) P + N + M + H, generating curated datasets denoted as P*, PN*, PNM*, and PNMH*, respectively. The workflow of the data curation is shown in Fig. 2.

We manually curated 520 lipophilicity measurements for molecules exceeding 700 Da, motivated by two key factors: first, there is a growing interest among medicinal chemists in large and structurally complex molecules (bRo5, see above); second, a previous study by Mannhold *et al*.^4^ have reported low predictive *logP* accuracy for these compounds.

Properties for these large molecules, including lipophilicity, remain far less systematically characterised than for classical small molecules. This lack of data, coupled with the fact that many bRo5 compounds display chameleonic behaviour — adopting distinct conformations in different environments and potentially leading to property cliffs — further complicates modelling. To address these issues and ensure high data quality, we carefully curated the available measurements by cross-checking sources and experimental conditions, without performing modelling. This focused process yielded an additional set of 284 unique compounds, which we retained separately.

The resulting dataset represents a comprehensive collection of experimentally determined values curated for Quantitative Structure-Activity Relationship (QSAR) modelling purposes. It should be noted, however, that a fraction of the compounds may not be represented at their highest level of stereochemical fidelity. While chemical names and CAS RN were collected directly from original publications and may indicate the presence of stereochemistry, the corresponding structural representations do not always explicitly encode this information. For QSAR modelling, this limitation is unlikely to significantly impact model performance; however, it represents an area where further curation efforts could enhance the quality of the dataset. We encourage the community to contribute to the continued refinement of this dataset, as improved stereochemical representation may be particularly relevant for applications where stereochemistry plays a critical role in biological activity.

## Data Record

*SangsterLogP* comprises 23,520 unique compounds and is provided in Microsoft Excel (XLSX) format^32^. It includes lipophilicity measurements and associated metadata, as detailed in Table 1.

**Table 1:** Column names, descriptions, and data types in the *SangsterLogP* dataset.

Column name	Description	Type
ID	Compound ID as reported in OCHEM	string
SMILES	Compound’s SMILES representation	string
Name	Compound name as reported in the source	string
Lipophilicity	Experimental partition or distribution coefficient (*logP* or *logD*)	float
pH	Experimental pH (when present)	float
ΔDP	Calculated ionisation-related shift (when applicable)	float
Ionisation-based class	The attributed ionisation class based on pH annotation and ΔDP	string
*logP* (exp or adj)	Experimental or adjusted logarithm of the partition coefficient	float
Adj pH	Optimal pH for major neutral species (when *logP* was adjusted)	float
Temperature	Experimental temperature in degrees Celsius (when present)	float
Exp method*	Technique used to measure *logP*/*logD* values	string
Recommended	“yes”: preferred value according to Dr Sangster	bool
Lipinski descriptors**	MW, MolLogP^37^, HBD, and HBA separated by semicolons	string
bRo5?	Binary indicator of bRo5 (MW > 500 and another violation or MW > 700)	bool
Source	DOI and PMID (when available), or APA citation of the source	string

*The methods’ codes (e.g., AS, HPLC) are as defined in Sangster^11^. When present, a hyphen separates the equilibration method from the quantification method.

**Calculated using RDKit (v. 2022.03.1).

## Technical Validation

We evaluated the improvement provided by our *SangsterLogP* dataset using two criteria: the extension of the covered chemical space and the consensus *logP* model validation performances.

### Extension of the covered chemical space

**Fig. 3:**
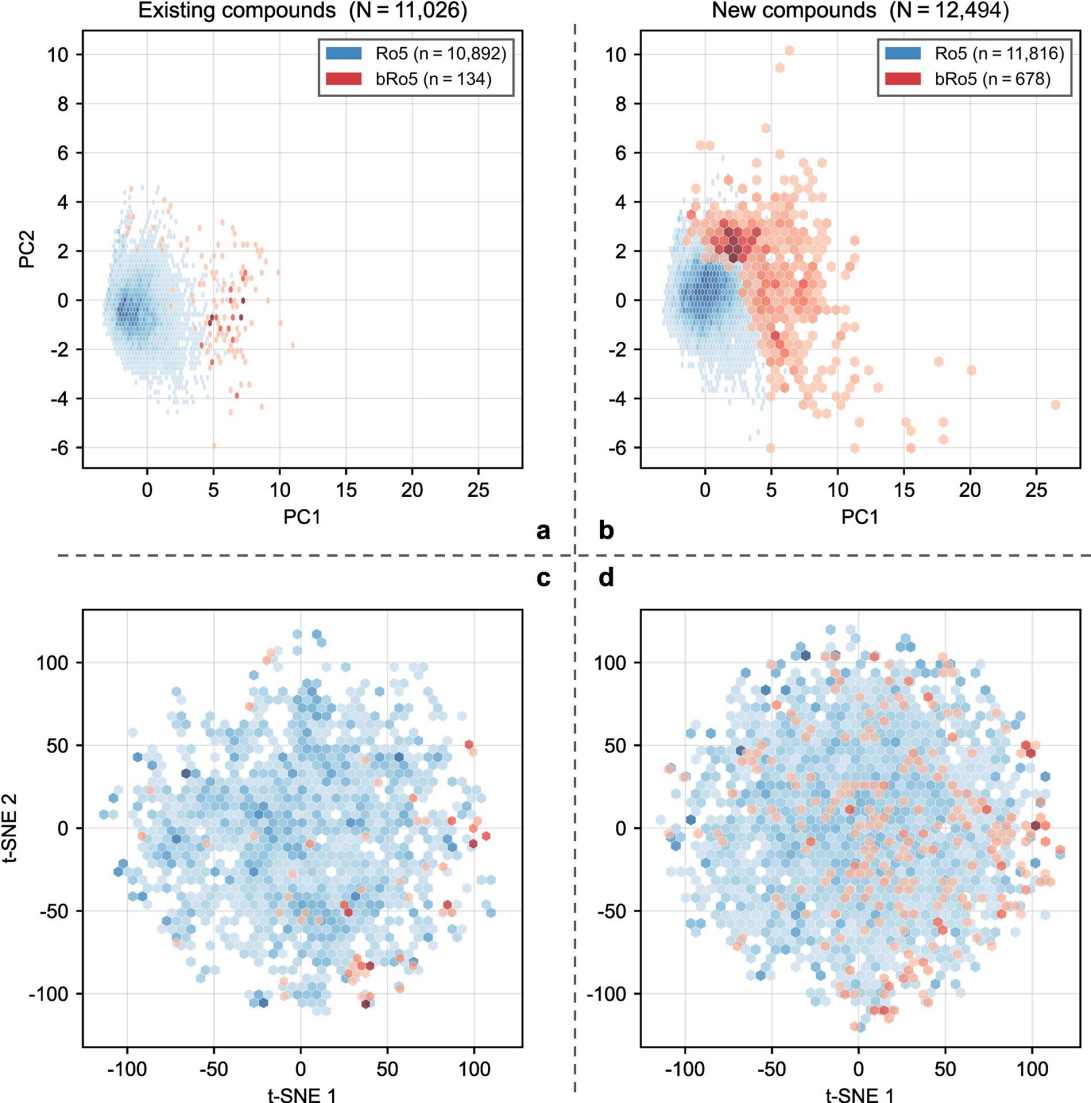
Chemical space distribution of *SangsterLogP* dataset compounds relative to PHYSPROP. Panels **a** and **c** show existing compounds (present in both datasets); panels **b** and **d** show new compounds (unique to SangsterLogP). Top panels (**a,b**) show PCA based on six physicochemical descriptors (molecular weight, Wildman–Crippen *log P*^37^, topological polar surface area, hydrogen bond acceptors, hydrogen bond donors, and number of rotatable bonds). Bottom panels (**c,d**) show t-SNE based on Morgan fingerprints (ECFP4). Blue hexagons represent Ro5-compliant molecules; red hexagons represent bRo5 molecules (MW > 500 Da with at least one additional Ro5 violation, or MW > 700 Da). Hexagon colour intensity indicates local molecular density.

To characterise the chemical space covered by our *SangsterLogP* dataset, we compared its physicochemical and structural diversity with that of the PHYSPROP collection^5^, previously curated and used as the training set for the ALogPS model^16^. For physicochemical diversity, we generated a low-dimensional projection of the chemical space using a principal component analysis (PCA) of the Lipinski Ro5^33^ and Veber’s rule^34^ descriptors. For structural diversity, we applied a *t*-Distributed Stochastic Neighbour Embedding (*t*-SNE) using Morgan circular fingerprints (radius = 2, 2048 bits). In Fig. 3, PCA and t-SNE analyses reveal the chemical space distribution of the *SangsterLogP* dataset in relation to the established PHYSPROP database. Left panels (a and c) show existing compounds — those already present in PHYSPROP — demonstrating the overlap between the two databases. Right panels (b and d) displays new compounds (those unique to our dataset). The visual difference in compound density between these panels reflects the broader chemical space coverage of the new compounds, with points distributed across a wider range of both physicochemical properties and chemical structure. This expansion indicates that *SangsterLogP* provides *logP* measurements for molecules with more diverse structural features and property combinations than previously available in PHYSPROP. Additionally, the new dataset shows a substantial expansion of bRo5 chemical space (shown in red), highlighting *SangsterLogP*’s enhanced coverage of larger, more complex molecular structures beyond traditional drug-like space.

#### Improvement in external validation

To assess the impact of data curation and the contribution of each confidence-stratified subset, we built *logP* consensus models using the same ML algorithms described in *Methods*, training them separately on each subset. The models’ performance was evaluated by 5-fold cross-validation (5CV) and two independent validation sets, and the results before and after curation were compared across all subsets (Table 2).

**Table 2:** Performance of models developed using the same protocol with different subsets.

Dataset	N	RMSE		
		5CV	Huuskonen set (N = 1,836)	Prospective set (N = 327)
P	23,588	0.560 ± 0.007	0.41 ± 0.01	0.48 ± 0.04
P*	14,547	0.422 ± 0.006	0.41 ± 0.01	0.43 ± 0.04
P + N	34,660	0.636 ± 0.006	0.35 ± 0.01	0.48 ± 0.02
PN*	22,279	0.455 ± 0.005	0.34 ± 0.01	0.43 ± 0.02
P + N + M	36,687	0.583 ± 0.005	0.34 ± 0.01	0.48 ± 0.02
PNM*	23,236	0.455 ± 0.004	0.33 ± 0.01	0.44 ± 0.02
P + N + M + H	42,854	0.660 ± 0.006	0.40 ± 0.02	0.56 ± 0.02
PNMH*	26,477	0.483 ± 0.004	0.36 ± 0.01	0.49 ± 0.02
***SangsterLogP***	23,520	0.456 ± 0.004	0.34 ± 0.01	0.47 ± 0.03

The first independent set included 145 *logP* measurements determined by Rains *et al*.^7^ as well as 182 compounds measured by Enamine^35^ (prospective validation). Both papers were published in 2025. The second set was from Huuskonen *et al*.^36^ and contained 1870 measurements from the same temporal window as the Sangster data (retrospective validation). After removing small molecules (≤3 atoms) and zwitterions, the Huuskonen set comprised 1,836 compounds. Using both validation sets ensured robust evaluation across temporal dimensions and allowed a more reliable assessment of the models’ accuracy across different *logP* datasets. To ensure rigorous validation, all chemical structures overlapping between our dataset and the validation sets were systematically identified and removed from the training data.

As shown in Table 2, data curation significantly improved model accuracy according to 5CV results. It also improved external prediction performance across both datasets in all but one case: models trained on the P/P* sets performed identically when predicting the Huuskonen set. The increase in diversity of chemical compounds in P -> N -> M - > H sets resulted in lower 5CV model performances, but up to the addition of subset M, it did not significantly affect the accuracy of models for both test sets. The inclusion of subset **H** increased both 5CV and test-set errors in PNMH*, and so our final curated set included only the PNM* subset and the additional 284 large molecules (MW > 700 Da) manually curated. As we can see, their addition only non-significantly increased 5CV and independent set prediction performance. Interestingly, the validation errors of models developed in this study for the Huuskonen full set (N = 1870) were lower (up to 0.41 log units) than the results originally reported for this set by the authors, *i.e*. RMSE = 0.46, which was calculated using the Leave-One-Out method^36^.

Overall, these analyses show that the expansion of the dataset achieved through careful correction and curation of data to construct the *SangsterLogP* dataset did not significantly affect the predictive performance. However, by incorporating structurally diverse and previously under-represented compounds — particularly in the bRo5 region — the resulting dataset provides broader coverage of chemical space, which is expected to support improved generalisation to independent benchmark sets. Importantly, the predictive errors across all curated subsets fall within the typical experimental uncertainty of lipophilicity measurements (approximately 0.3–0.5 log units)^3,19^, indicating that the enhanced dataset enables models to reach the practical accuracy limits imposed by the underlying experimental variability.

## Data Availability

The *SangsterLogP* dataset presented in this *data descriptor* is publicly available in an XLSX file via Zenodo (10.5281/zenodo.19387551)^32^. The repository also includes the test sets used in the benchmark analysis in the same file. In addition, the data are implemented within the OCHEM (https://ochem.eu/article/161956) to support visualisation and modelling.

## Code availability

The Python v.3.12.8 script used to implement the dataset classification and the *logD*-to-*logP* correction described in the *Methods* is available on GitHub and has been archived on Zenodo (10.5281/zenodo.19625460)^20^. Third-party software included ChemAxon Calculator Plugins v.5.10.4 (https://chemaxon.com) and OCHEM (https://ochem.eu).

## References

[1] Ratkova, E. L. *et al*. Empirical and physics-based calculations of physical-chemical properties. in *Reference Module in Chemistry, Molecular Sciences and Chemical Engineering B9780443298080000583*. 10.1016/B978-0-443-29808-0.00058-3 (Elsevier, 2025).

[2] Shultz, M. D. Two Decades under the Influence of the Rule of Five and the Changing Properties of Approved Oral Drugs: Miniperspective. *J. Med. Chem.* **62**, 1701–1714, 10.1021/acs.jmedchem.8b00686 (2019). 30212196 10.1021/acs.jmedchem.8b00686

[3] Lombardo, F., Faller, B., Shalaeva, M., Tetko, I. & Tilton, S. The Good, the Bad and the Ugly of Distribution Coefficients: Current Status, Views and Outlook. in *Methods and Principles in Medicinal Chemistry* (ed. Mannhold, R.) 407–437. 10.1002/9783527621286.ch16 (Wiley, 2007).

[4] Mannhold, R., Poda, G. I., Ostermann, C. & Tetko, I. V. Calculation of molecular lipophilicity: State-of-the-art and comparison of log P methods on more than 96,000 compounds. *J Pharm Sci* **98**, 861–893, 10.1002/jps.21494 (2009). 18683876 10.1002/jps.21494

[5] Howard, P. & Meylan, W. *Physical/Chemical Property Database (PHYSPROP)*. (Syracuse Research Corporation, Environmental Science Center North Syracuse NY, 1999).

[6] Hansch, C., Leo, A. & Hoekman, D. H. *Exploring QSAR*. (ACS, Washington, 1995).

[7] Rains, J., Steeples, E., Robinson, B., Pierce, A. J. & Sayer, D. High-Throughput HPLC Method for the Measurement of Octanol–Water Partition Coefficients without an Organic Modifier. *Anal. Chem.* **97**, 12321–12328, 10.1021/acs.analchem.5c01411 (2025). 40473576 10.1021/acs.analchem.5c01411

[8] Wenlock, M. & Tomkinson, N. Experimental *in vitro* DMPK and physicochemical data on a set of publicly disclosed compounds. *ChEMBL*10.6019/CHEMBL3301361 (2015).

[9] Tetko, I. V., M. Lowe, D. & Williams, A. J. The development of models to predict melting and pyrolysis point data associated with several hundred thousand compounds mined from PATENTS. *J Cheminform* **8**, 2, 10.1186/s13321-016-0113-y (2016). 26807157 10.1186/s13321-016-0113-y PMC4724158

[10] Doak, B. C., Over, B., Giordanetto, F. & Kihlberg, J. Oral Druggable Space beyond the Rule of 5: Insights from Drugs and Clinical Candidates. *Chemistry & Biology* **21**, 1115–1142, 10.1016/j.chembiol.2014.08.013 (2014). 25237858 10.1016/j.chembiol.2014.08.013

[11] Sangster, J. Octanol-Water Partition Coefficients of Simple Organic Compounds. *Journal of Physical and Chemical Reference Data* **18**, 1111–1229, 10.1063/1.555833 (1989).

[12] Tetko, I. V. *et al*. Virtual Computational Chemistry Laboratory – Design and Description. *J Comput Aided Mol Des* **19**, 453–463, 10.1007/s10822-005-8694-y (2005). 16231203 10.1007/s10822-005-8694-y

[13] Sushko, I. *et al*. Online chemical modeling environment (OCHEM): web platform for data storage, model development and publishing of chemical information. *J Comput Aided Mol Des* **25**, 533–554, 10.1007/s10822-011-9440-2 (2011). 21660515 10.1007/s10822-011-9440-2 PMC3131510

[14] Sushko, I., Salmina, E., Potemkin, V. A., Poda, G. & Tetko, I. V. ToxAlerts: A Web Server of Structural Alerts for Toxic Chemicals and Compounds with Potential Adverse Reactions. *J. Chem. Inf. Model.* **52**, 2310–2316, 10.1021/ci300245q (2012). 22876798 10.1021/ci300245q PMC3640409

[15] Tetko, I. V. & Poda, G. I. Application of ALOGPS 2.1 to predict log D distribution coefficient for Pfizer proprietary compounds. *J Med Chem* **47**, 5601–5604, 10.1021/jm049509l (2004). 15509156 10.1021/jm049509l

[16] Tetko, I. V., Tanchuk, V. Y. & Villa, A. E. Prediction of n-octanol/water partition coefficients from PHYSPROP database using artificial neural networks and E-state indices. *J Chem Inf Comput Sci* **41**, 1407–1421, 10.1021/ci010368v (2001). 11604042 10.1021/ci010368v

[17] Tetko, I. V. & Tanchuk, V. Y. Application of associative neural networks for prediction of lipophilicity in ALOGPS 2.1 program. *J Chem Inf Comput Sci* **42**, 1136–1145, 10.1021/ci025515j (2002). 12377001 10.1021/ci025515j

[18] Tetko, I. V. & Bruneau, P. Application of ALOGPS to predict 1-octanol/water distribution coefficients, logP, and logD, of AstraZeneca in-house database. *J Pharm Sci* **93**, 3103–3110, 10.1002/jps.20217 (2004). 15514985 10.1002/jps.20217

[19] Lombardo, F., Shalaeva, M. Y., Tupper, K. A. & Gao, F. ElogD_oct_: A Tool for Lipophilicity Determination in Drug Discovery. 2. Basic and Neutral Compounds. *J. Med. Chem.* **44**, 2490–2497, 10.1021/jm0100990 (2001). 11448232 10.1021/jm0100990

[20] Cirino, T. SangsterLogP (v1.0). *Zenodo*10.5281/ZENODO.19625460 (2026).

[21] Karpov, P., Godin, G. & Tetko, I. V. Transformer-CNN: Swiss knife for QSAR modeling and interpretation. *J Cheminform* **12**, 17, 10.1186/s13321-020-00423-w (2020). 33431004 10.1186/s13321-020-00423-w PMC7079452

[22] Makarov, D. M., Fadeeva, Y. A., Shmukler, L. E. & Tetko, I. V. Beware of proper validation of models for ionic Liquids! *Journal of Molecular Liquids* **344**, 117722, 10.1016/j.molliq.2021.117722 (2021).

[23] Reiser, P., Eberhard, A. & Friederich, P. Graph neural networks in TensorFlow-Keras with RaggedTensor representation (kgcnn). *Software Impacts* **9**, 100095, 10.1016/j.simpa.2021.100095 (2021).

[24] Xiong, Z. *et al*. Pushing the Boundaries of Molecular Representation for Drug Discovery with the Graph Attention Mechanism. *J. Med. Chem.* **63**, 8749–8760, 10.1021/acs.jmedchem.9b00959 (2020). 31408336 10.1021/acs.jmedchem.9b00959

[25] Heid, E. *et al*. Chemprop: A Machine Learning Package for Chemical Property Prediction. *J. Chem. Inf. Model.* **64**, 9–17, 10.1021/acs.jcim.3c01250 (2024). 38147829 10.1021/acs.jcim.3c01250 PMC10777403

[26] Hall, L. H. & Kier, L. B. Electrotopological State Indices for Atom Types: A Novel Combination of Electronic, Topological, and Valence State Information. *J. Chem. Inf. Comput. Sci.* **35**, 1039–1045, 10.1021/ci00028a014 (1995).

[27] Sosnin, S., Karlov, D., Tetko, I. V. & Fedorov, M. V. Comparative Study of Multitask Toxicity Modeling on a Broad Chemical Space. *J. Chem. Inf. Model.* **59**, 1062–1072, 10.1021/acs.jcim.8b00685 (2019). 30589269 10.1021/acs.jcim.8b00685

[28] Gerstein, S. & Godin, G. Osmordred: Unified RDkit new descriptors in c++. *GitHub* https://github.com/osmoai/osmordred (2025).

[29] Prokhorenkova, L., Gusev, G., Vorobev, A., Dorogush, A. V. & Gulin, A. CatBoost: unbiased boosting with categorical features. *Advances in neural information processing systems* **31**, 10.48550/ARXIV.1706.09516 (2018).

[30] Tetko, I. V. Tox24 Challenge. *Chem. Res. Toxicol.* **37**, 825–826, 10.1021/acs.chemrestox.4c00192 (2024). 38769907 10.1021/acs.chemrestox.4c00192

[31] Tetko, I. V. *et al*. Critical assessment of QSAR models of environmental toxicity against Tetrahymena pyriformis: focusing on applicability domain and overfitting by variable selection. *J Chem Inf Model* **48**, 1733–1746, 10.1021/ci800151m (2008). 18729318 10.1021/ci800151m

[32] Cirino, T., Charochkina, L., Tetko, I. & Sangster, J. SangsterLogP dataset. *Zenodo*10.5281/ZENODO.19387551 (2026).

[33] Lipinski, C. A., Lombardo, F., Dominy, B. W. & Feeney, P. J. Experimental and computational approaches to estimate solubility and permeability in drug discovery and development settings. *Advanced Drug Delivery Reviews* **23**, 3–25, 10.1016/S0169-409X(96)00423-1 (1997). 10.1016/s0169-409x(00)00129-0 11259830

[34] Veber, D. F. *et al*. Molecular Properties That Influence the Oral Bioavailability of Drug Candidates. *J. Med. Chem.* **45**, 2615–2623, 10.1021/jm020017n (2002). 12036371 10.1021/jm020017n

[35] Gurbych, O. *et al*. Filling the Gap in LogP and pKa Evaluation for Saturated Fluorine‐Containing Derivatives With Machine Learning. *J Comput Chem* **46**, e70002, 10.1002/jcc.70002 (2025). 39803824 10.1002/jcc.70002

[36] Huuskonen, J. J., Livingstone, D. J. & Tetko, I. V. Neural Network Modeling for Estimation of Partition Coefficient Based on Atom-Type Electrotopological State Indices. *J. Chem. Inf. Comput. Sci.* **40**, 947–955, 10.1021/ci9904261 (2000). 10955523 10.1021/ci9904261

[37] Wildman, S. A. & Crippen, G. M. Prediction of Physicochemical Parameters by Atomic Contributions. *J. Chem. Inf. Comput. Sci.* **39**, 868–873, 10.1021/ci990307l (1999).

